# Distinct Somatic Discrimination Reflected by Laser-Evoked Potentials Using Scalp EEG Leads

**DOI:** 10.1007/s40846-016-0159-3

**Published:** 2016-08-11

**Authors:** Jen-Jui Hsueh, Jia-Jin Jason Chen, Fu-Zen Shaw

**Affiliations:** 1 Institute of Biomedical Engineering, National Cheng Kung University, Tai-nan, 701 Taiwan; 2 Department of Psychology, National Cheng Kung University, 1 University Road, Tai-nan, 701 Taiwan; 3 Mind Research and Imaging Center, National Cheng Kung University, Tai-nan, 701 Taiwan

**Keywords:** Laser-evoked potential (LEP), Pain, Equivalent dipole, Primary somatosensory cortex, Secondary somatosensory cortex

## Abstract

Discrimination is an important function in pain processing of the somatic cortex. The involvement of the somatic cortex has been studied using equivalent dipole analysis and neuroimaging, but the results are inconsistent. Scalp electroencephalography (EEG) can reflect functional changes of particular brain regions underneath a lead. However, the responses of EEG leads close to the somatic cortex in response to pain have not been systematically evaluated. The present study applied CO_2_ laser stimulation to the dorsum of the left hand. Laser-evoked potentials (LEPs) of C4, T3, and T4 leads and pain ratings in response to four stimulus intensities were analyzed. LEPs started earlier at the C4 and T4 leads. The onset latency and peak latency of LEPs for C4 and T4 leads were the same. Only 10 of 22 subjects (45 %) presented equivalent current dipoles within the primary somatosensory or motor cortices. LEP amplitudes of these leads increased as stimulation intensity increased. The stimulus–response pattern of the C4 lead was highly correlated with pain rating. In contrast, an S-shaped stimulus–response curve was obtained for the T3 and T4 leads. The present study provides supporting evidence that particular scalp channels are able to reflect the functional characteristics of their underlying cortical areas. Our data strengthen the clinical application of somatic-cortex-related leads for pain discrimination.

## Introduction

Pain perception has a protective role that prevents injury. An individual learns to produce immediate aversive behavior to keep away from harmful stimuli. An important function of the pain process is discrimination and localization of a nociceptive stimulus [1]. A lesion in the postcentral gyrus causes problems in sensory discrimination or asomaesthesia in humans [2, 3] and animals [4]. Effected individuals cannot locate or characterize a noxious stimulus well, and are thus at risk for severe injury.

Although several brain areas have been found to be involved in the pain process in neuroimaging studies [5, 6], the results are inconsistent, especially for the primary somatosensory cortex (S1). Discrimination or detection of a painful stimulus is crucial for clinic evaluation. Of the available tools for exploring brain function, electroencephalography (EEG) has the advantages of portability, channel selectivity, fine temporal resolution, easy integration with other apparatus, safety, relatively low cost. Scalp EEG leads are usually used to examine brain function [7]. Some leads, such as Cz and Pz, are clinically used to acquire pain-related responses [8–10]. These EEG recordings focus on a midline brain region. However, few studies have been conducted on the discrimination of somatosensory regions, such as S1 and the secondary somatosensory cortex (S2) [11, 12], using scalp EEG leads (T3/T4 or C3/C4). Equivalent dipole analysis is commonly used to explore the roles of S1 and S2 in pain, but the results are inconsistent [13–15]. A simple method for determining the functional roles of S1 and S2 in the pain process is desirable.

Laser stimulation is a non-invasive tool that can be easily applied to a wide body area. A CO_2_ laser can produce high energy to activate nociceptors simultaneously and exclusively [16]. Laser-evoked potential (LEP) can be used to understand the pain process of the brain in healthy subjects and patients [9, 17, 18]. The results of equivalent dipole analysis of LEPs are inconsistent in somatic regions [13, 19–21]. In addition, such analysis is time-consuming. In general, scalp EEG can reflect the neural activities of brain regions underneath a lead [22]. Therefore, the LEPs of particular scalp leads, such as T3/T4 or C3/C4, can be used to understand the somatic process of pain. However, pain discrimination of somatosensory cortex-related leads has not been well investigated using LEPs under various laser intensities.

The present study tests the hypothesis that directly measuring LEPs using scalp EEG leads can reflect distinct intensity discrimination within the somatic cortical areas. The stimulus–response pattern in terms of a subjective measure (pain rating) and objective measures (LEP amplitudes in the C4, T4, and T3 leads) was obtained. We anticipated that the onset latency and peak latency of LEPs between the three leads would be different based on inter- and intra-hemispheric pain processes under four stimulus intensities. LEP amplitudes of these leads increased as stimulation intensity increased with different stimulus–response patterns, indicating the distinct cortical process of pain.

## Materials and Methods

### Participants

Thirty-two healthy right-handed volunteers (23.1 ± 2.3 years old, range 20–30 years, 18 males and 14 females) were enrolled in this study. Ten participants engaged in an experiment of determining the pain threshold based on laser stimulation. Twenty-two subjects participated in an experiment of subjective feeling and objective cortical activities in response to various laser intensities. Written informed consent was given by all participants. The experimental procedure was reviewed and approved by a local Research Ethics Committee.

### Laser Stimulation and Pain Rating

Cutaneous noxious stimuli were generated by an infrared CO_2_ laser stimulator with a 10.6-µm wavelength (Blue Sky Tech Co., Ltd., Taiwan). The stimulation site of the CO_2_ laser was indicated by a helium–neon laser. The laser was operated in TEM_00_ mode. The pulse duration and unfocused beam diameter were 30 ms and 2.5 mm, respectively. In our pilot study (n = 10), the pain threshold was set to 2 W (≥3 W produced a pin-prick-like pain sensation for five stimuli).

Four intensities, namely 50 % (1 W, 6.12 mJ/mm^2^), 100 % (2 W, 12.23 mJ/mm^2^), 150 % (3 W, 18.34 mJ/mm^2^), and 200 % (4 W, 24.46 mJ/mm^2^) of the pain threshold, were used in the following experiment. To minimize possible tissue damage and reduce possible sensitization or habituation, the stimuli were randomly applied to an area of 5 × 5 cm^2^ over the dorsum of the left hand. The inter-stimulus interval was randomly varied between 7 and 14 s. The four laser intensities did not cause any visible damage to the skin throughout the entire experiment.

Participants were seated comfortably with eyes open in a quiet room. They wore protective goggles for safety and ear plugs to prevent any acoustic interference from the laser device. The degree of pain perception was determined using a visual analogous scale (VAS). The VAS consisted of a 100-mm horizontal line with “no pain at all” (score 0) on one end and “extreme pain” (score 100) on the other end. The VAS has been validated for subjective pain measure [23]. Participants were asked to pay attention to the dorsum of their left hand for each stimulus to ensure similar cognitive/attentive states [24]. After each stimulus, participants rated their pain using VAS. For example, the subjective perception of the threshold stimulation was ~5 on VAS in our first experiment. A set of 20 stimuli was conducted before LEP recordings, which allowed the participants to become familiar with the experimental procedure. Subsequently, a total of 240 laser stimuli (60 for each intensity) were applied for all participants (n = 22) with a random order of the four laser intensities.

### LEP Recording and Analysis

EEG data were recorded using a 40-channel amplifier (NuAmps, Compumedics Ltd., VIC, Australia) through a 32-channel Ag/AgCl electrode cap. The electrode arrangement of the cap was based according to the international 10–20 system with reference to link bilateral mastoid processes. The impedance of each electrode was kept at below 5 kΩ. The sampling rate for data acquisition was 500 Hz. Raw EEG data were band-pass-filtered between 0.5 and 30 Hz.

LEP epochs were extracted with a period containing a pre-stimulus segment of 100 ms and a post-stimulus segment of 600 ms. Epochs contaminated with blinks, eye-movement artifacts (>65 μV), or remarkable muscle activity were excluded. Time-locked averaged LEPs with regard to the laser stimulus were recorded. According to the EEG montage with reference to neuroanatomy [22], the C4, T4, and T3 leads were nearest to contralateral S1 (cS1), contralateral S2 (cS2), and ipsilateral S2 (iS2), respectively. In the present study, averaged LEPs of the C4, T4, and T3 leads were further analyzed.

**Fig. 1:**
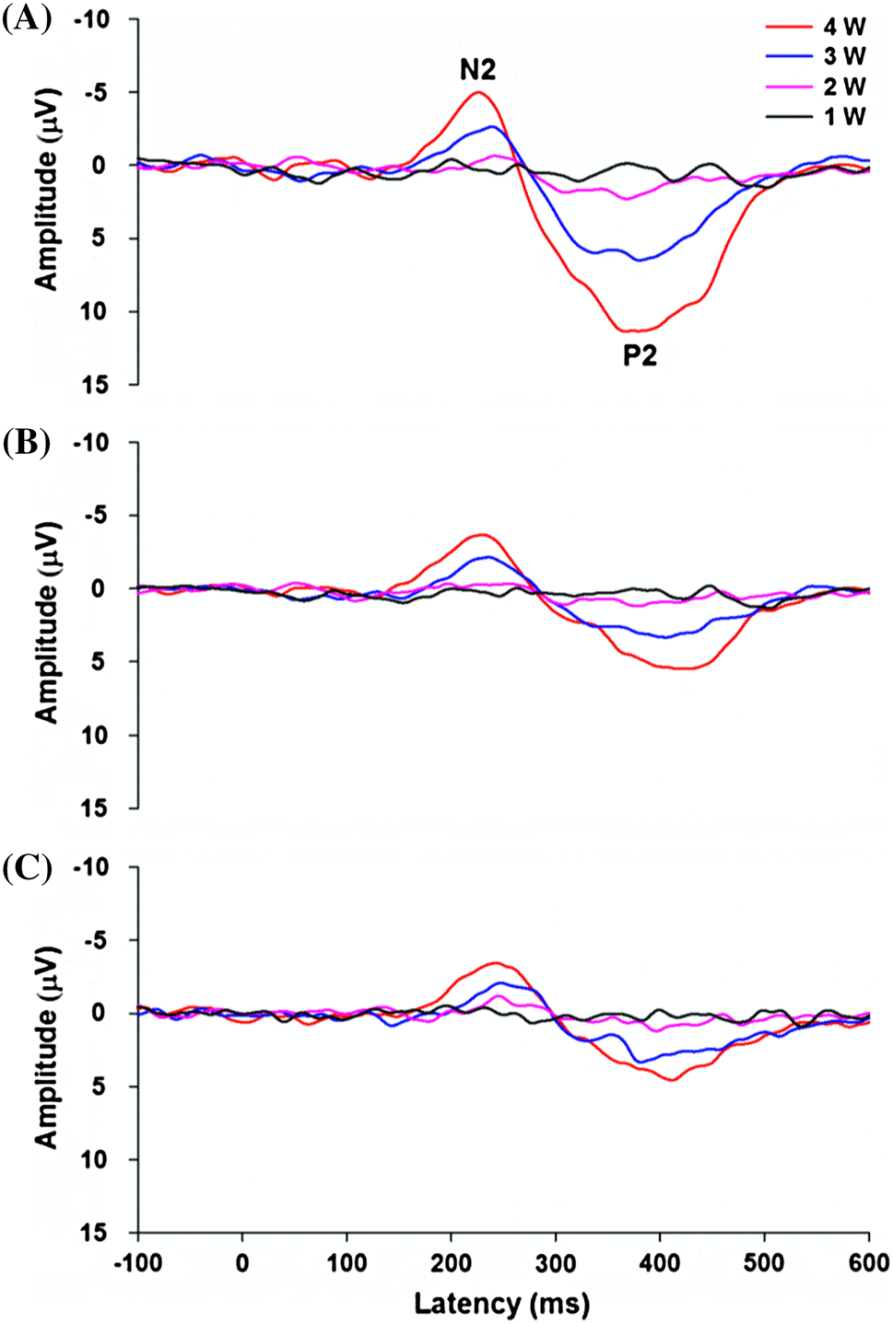
Grand averaged LEPs for four stimulus intensities in **(A)** C4, **(B)** T4, and **(C)** T3 leads. Maximal negative *peak* and positive *peak* of LEPs are labeled *N2* and *P2*, respectively. No clear response is observed under 1-W stimulus intensity

For averaged LEPs, the largest and mostly negative peak was defined as N2, and the largest subsequent positive peak was defined as P2 (Fig. 1). In general, time windows of ±60 ms with respect to the N2 or P2 peak of the 4-W LEP were accepted for identifying cortical responses. The N2–P2 peak-to-peak amplitudes of three-channel LEPs in response to the four laser intensities were calculated. Both the peak latency and onset latency of LEPs for the C4, T4, and T3 leads were measured. The grand average of LEPs across all subjects was calculated.

### Spatiotemporal Analysis of LEPs

Scalp topographic maps of LEPs were analyzed using MATLAB (The Mathworks, Inc., Natick, MA) and the open source toolbox EEGLAB (Swartz Center for Computational Neuroscience, La Jolla, CA). Contour plots of the grand average of LEPs were created with intervals of 30 ms.

To further understand the possible source distribution of LEPs, the spatiotemporal source model was used to assess equivalent current dipoles of LEPs using Curry 7 software (NeuroScan, Inc., USA) [18]. Regional dipole fitting was used to fit the signal. The boundary element method for a realistic head model based on the MNI averaged structure image was used. According to previous reports on the dipole source analysis of LEPs [18, 19], a four-dipole model was calculated. Residual variance is the percentage of data that cannot be explained by the model. During the optimization process for the four-dipole model of LEPs, a criterion for the residue variance of <10 % was used [19]. In the present study, 4-W LEPs were selected for dipole analysis because they were the greatest and consistently appeared for all subjects.

### Functional Correlation Analysis

To investigate the functional correlates of subjective VAS values and objective amplitudes of three-channel LEPs in response to the four laser intensities, two regression methods were used. Both subjective VAS values and peak-to-peak amplitudes of three-channel LEPs were normalized by their values in response to a 4-W stimulation. Following a previous study [25], the relations between normalized LEP amplitudes of each individual channel (C4, T4, and T3) and normalized VAS with regard to four laser intensities were determined for each participant using exponential curve fitting ($f(x) = a_{0}\,*\,e^{{(a_{1}\,*\,x)}}$).

To further study the stimulus–response pattern, the polynomial function *f*(*x*) = *b*_0_ + (*b*_1_ × *x*) + (*b*_2_ × *x*^2^) + (*b*_3_ × *x*^3^) was used for normalized amplitudes of three-channel LEPs and normalized VAS values in response to the four laser intensities. Coefficient b_1_ indicates a linear increase (slope). Coefficients b_2_ and b_3_ reflect the shape pattern of the fitted curve. For example, higher values of b_2_ and b_3_ indicate a tendency of nonlinearity.

### Statistical Analysis

Two-factor repeated measure analysis of variance followed by post hoc comparisons with Bonferroni corrections were conducted on VAS values, peak-to-peak amplitudes of LEPs, and onset latencies and peak latencies of LEPs. R^2^ (goodness of fit) values were calculated for the exponential fitting for normalized peak-to-peak amplitudes of LEPs and VAS values in response to the four laser intensities. A paired *t* test was conducted on the R^2^ values of exponential curve fitting, change of normalized LEP amplitude between 2- and 3-W stimulation, and coefficients (b_2_ and b_3_) of the polynomial function. The statistical analyses were performed using Statistical Package for Social Sciences version 17.0 software (SPSS, Chicago, IL). Data are expressed as the mean ± standard error of the mean. The two-tailed significance level was set at *p* ≤ 0.05.

## Results

### LEP Characteristics

Figure 1 illustrates LEPs in response to the four laser intensities (1, 2, 3, and 4 W). The first negative and positive peaks of LEPs were characterized as N2 and P2, respectively. No clear response was observed under the 1-W stimulus intensity. For the 2-W stimulation (pain threshold), 16 of 22 subjects (72.7 %) showed an obvious N2–P2 response at the C4 channel, and 9 subjects (40.9 %) showed an N2–P2 pattern at the T4 and T3 channels. All participants showed clear LEPs for 3- and 4-W stimulations.

LEP amplitudes increased as stimulus intensity increased (Fig. 1). The N2–P2 amplitudes of LEPs had a significant difference in terms of channel (*F*_2,42_ = 93.007, *p* < 0.001), intensity (*F*_3,63_ = 127.046, *p* < 0.001), and their interaction (*F*_6,126_ = 26.751, *p* < 0.001). The LEP amplitude at the C4 lead was significantly higher than those of the T3 and T4 leads for all stimulus intensities (Table 1). In addition, VAS values in response to 1–4 W stimulations were 3.04 ± 0.57, 11.85 ± 1.80, 24.99 ± 2.93, and 41.30 ± 4.04, respectively. The VAS values in response to the four stimulation intensities were significantly different (*F*_3,63_ = 85.877, *p* < 0.001). In general, the pain ratings increased as stimulation intensity increased.

**Table 1:** Latencies and LEP amplitudes of C4, T4, and T3 channels

	C4	T4	T3
Onset latency (ms)			
2 W	200.5 ± 5.6	196.0 ± 7.3	209.8 ± 10.1*^#^
3 W	169.6 ± 4.1^+^	168.5 ± 3.7^+^	189.4 ± 4.8^+^*^#^
4 W	153.0 ± 4.4^+$^	150.8 ± 4.0^+$^	171.0 ± 4.4^+$^*^#^
N2 latency (ms)			
2 W	249.5 ± 5.0	251.5 ± 8.9	251.6 ± 8.1
3 W	235.6 ± 3.8^+^	232.1 ± 4.2^+^	253.0 ± 4.3*^#^
4 W	225.7 ± 3.0^+$^	222.6 ± 4.6^+^	243.5 ± 4.0*^#^
P2 latency (ms)			
2 W	360.6 ± 8.8	362.2 ± 9.8	368.4 ± 9.9
3 W	367.5 ± 8.6	370.8 ± 8.4	369.1 ± 9.7
4 W	363.3 ± 7.8	369.1 ± 9.7	360.8 ± 9.0
LEP amplitude (μV)			
2 W	4.51 ± 0.68	1.63 ± 0.45*	1.56 ± 0.46*
3 W	14.85 ± 1.42^+^	8.24 ± 0.62^+^*	8.27 ± 0.79^+^*
4 W	22.75 ± 1.95^+$^	11.87 ± 0.82^+$^*	10.69 ± 0.95^+^*

* *p* < 0.05 versus C4, ^#^ *p* < 0.05 versus T4, ^+^ *p* < 0.05 versus 2 W, ^$^ *p* < 0.05 versus 3 W

In addition to the analysis of N2–P2 amplitudes of LEPs, the onset and peak latencies of LEPs from three scalp channels in response to three laser intensities were also analyzed (Table 1). The onset latency of LEPs had a significant difference in terms of channel (*F*_2,30_ = 14.942, *p* < 0.001) and intensity (*F*_2,30_ = 43.547, *p* < 0.001). The onset of LEPs occurred significantly earlier as stimulus intensity increased. The onset latency of LEPs at the ipsilateral T3 lead was significantly longer than those of the contralateral C4 and T4 leads. The N2 latency of LEPs had a significant difference in terms of channel (*F*_2,30_ = 4.212, *p* = 0.023) and intensity (*F*_2,30_ = 10.568, *p* < 0.001). The N2 peaks of the C4 and T4 leads occurred significantly earlier as stimulus intensity increased. The N2 latency of the ipsilateral T3 lead was significantly longer than those of the contralateral C4 and T4 leads at 3- and 4-W stimuli. The P2 latency of LEPs did not reveal a significant difference.

### Spatiotemporal Analysis of LEPs

**Fig. 2:**
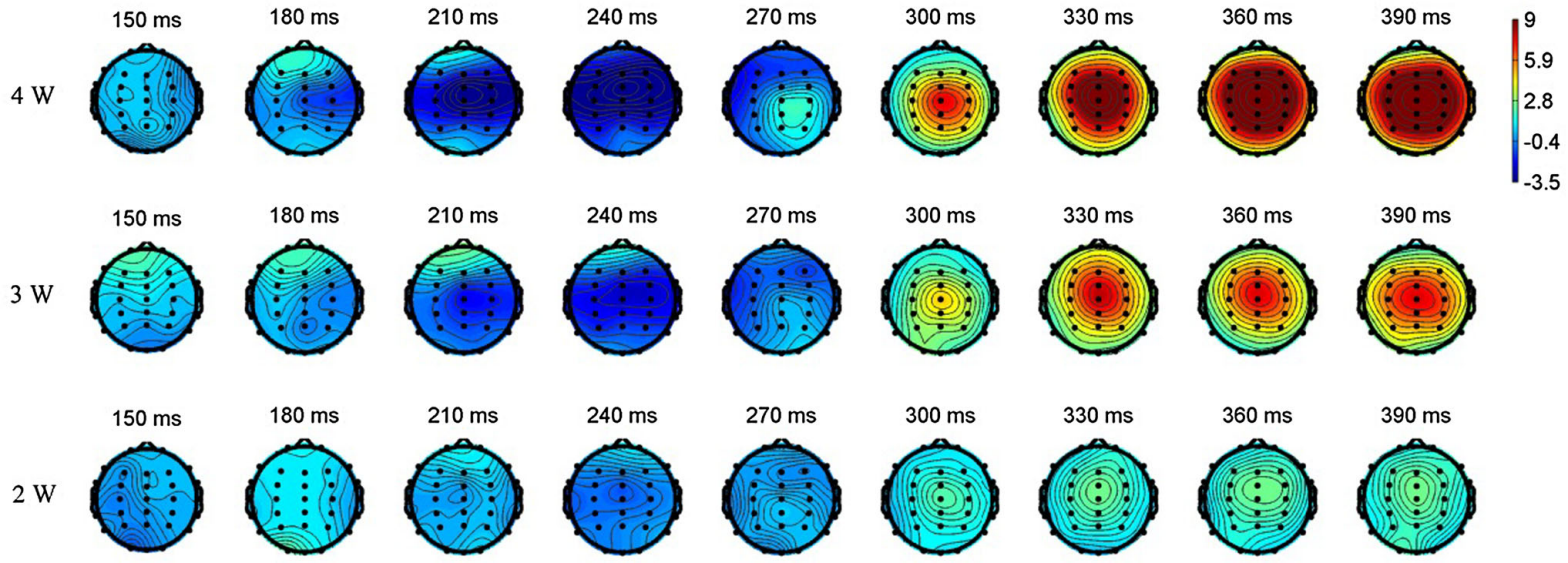
Topographic *map* of grand averaged LEPs under 2-, 3-, or 4-W stimulation. LEPs show concentric-like pattern at 180–210 ms over contralateral temporal–parietal region. LEPs at 300–390 ms reveal large positivity values over central region

Figure 2 shows topographic maps of LEPs over the scalp under three stimulus intensities (2, 3, and 4 W). A concentric contour appeared at 180–210 ms of LEPs and started at the contralateral temporal–parietal region, i.e., the C4-likelihood region. The N2 peak of LEPs also showed a similar contour around the C4 region (240–270 ms). The P2 peak of LEPs showed the maximal amplitude in the central region.

**Fig. 3:**
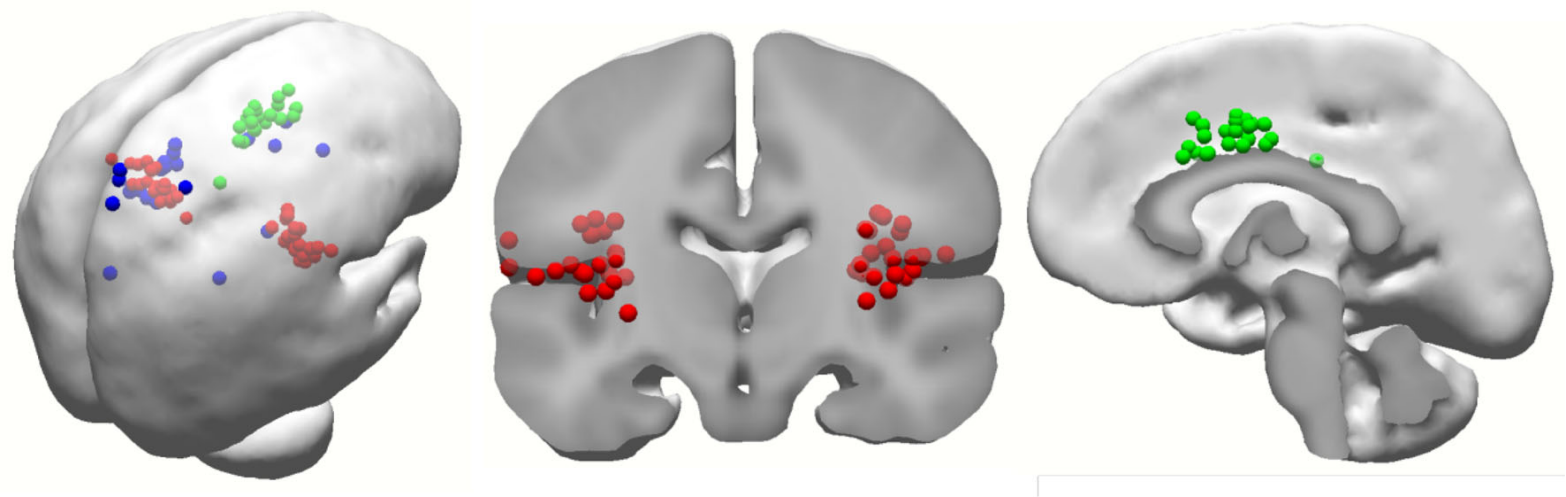
Distribution of all equivalent current dipoles of 4-W LEPs. S2/insula or neighborhood areas are characterized by *red dots*. Midline cortical dipoles, including anterior and middle cingulate gyri, are characterized by *green dots*. Contralateral dipoles within dorsal sensorimotor cortex, including primary somatosensory cortex and primary motor cortex, are characterized by *blue dots*

In addition to the scalp maps of LEPs, the equivalent current dipoles of 4-W LEPs were calculated using the spatiotemporal source model with four dipoles. In the present study, residual variance for dipole approximation was 6.47 ± 0.33 %. The dipoles are primarily located within the contralateral sensorimotor area, cingulate cortex, and bilateral S2 or insula (Fig. 3). The detailed coordinates and distribution of the dipoles are summarized in Table 2. All participants showed dipoles in the medial cortical region (64 % in the anterior cingulate cortex, 36 % in the middle cingulate cortex). 82 % of the equivalent current dipoles were located in the contralateral parasylvian region (50 % in S2, 32 % in the insula). Similar results were obtained for the ipsilateral parasylvian region (50 % in S2, 32 % in the insula). In contrast, the fourth equivalent current dipole was more widespread. In contrast to the highly consistent dipole locations for the previous three equivalent current sources, only 45 % of the fourth equivalent current dipoles were located in the sensorimotor region [27 % in S1, 18 % in the primary motor cortex (M1)]. Because of diverse distribution of sensory-cortex-related dipoles, the present study used LEP amplitudes of selected channels for further analysis instead of dipole strengths.

**Table 2:** MNI coordinates and number of dipoles in brain area

Brain areas	MNI coordinates			N (%)
	x (mm)	y (mm)	z (mm)	
Contralateral sensorimotor				10 (45)
S1	20.2 ± 7.3	−46.7 ± 5.6	71.5 ± 3.9	6 (27)
M1	16.5 ± 0.4	−29.0 ± 3.5	65.1 ± 1.4	4 (18)
Contralateral parasylvian				18 (82)
S2	45.7 ± 6.1	−17.2 ± 1.3	17.0 ± 3.6	11 (50)
Insula	34.3 ± 3.9	−18.6 ± 1.1	10.7 ± 3.3	7 (32)
Ipsilateral parasylvian				18 (82)
S2	−46.4 ± 9.2	−17.8 ± 1.5	16.2 ± 4.7	11 (50)
Insula	−33.3 ± 2.1	−18.3 ± 1.3	10.7 ± 5.2	7 (32)
Medial parts				22 (100)
ACC	1.6 ± 1.8	18.8 ± 5.8	29.7 ± 5.2	14 (64)
MCC	2.1 ± 1.7	3.5 ± 8.2	34.7 ± 2.9	8 (36)

*N* (*%*) number (proportion) of dipoles in brain area, *S1* primary somatosensory cortex, *M1* primary motor cortex, *S2* secondary somatosensory cortex, *ACC*, anterior cingulate cortex, *MCC* middle cingulate cortex

### Functional Correlations Between LEP Amplitudes and VAS

**Fig. 4:**
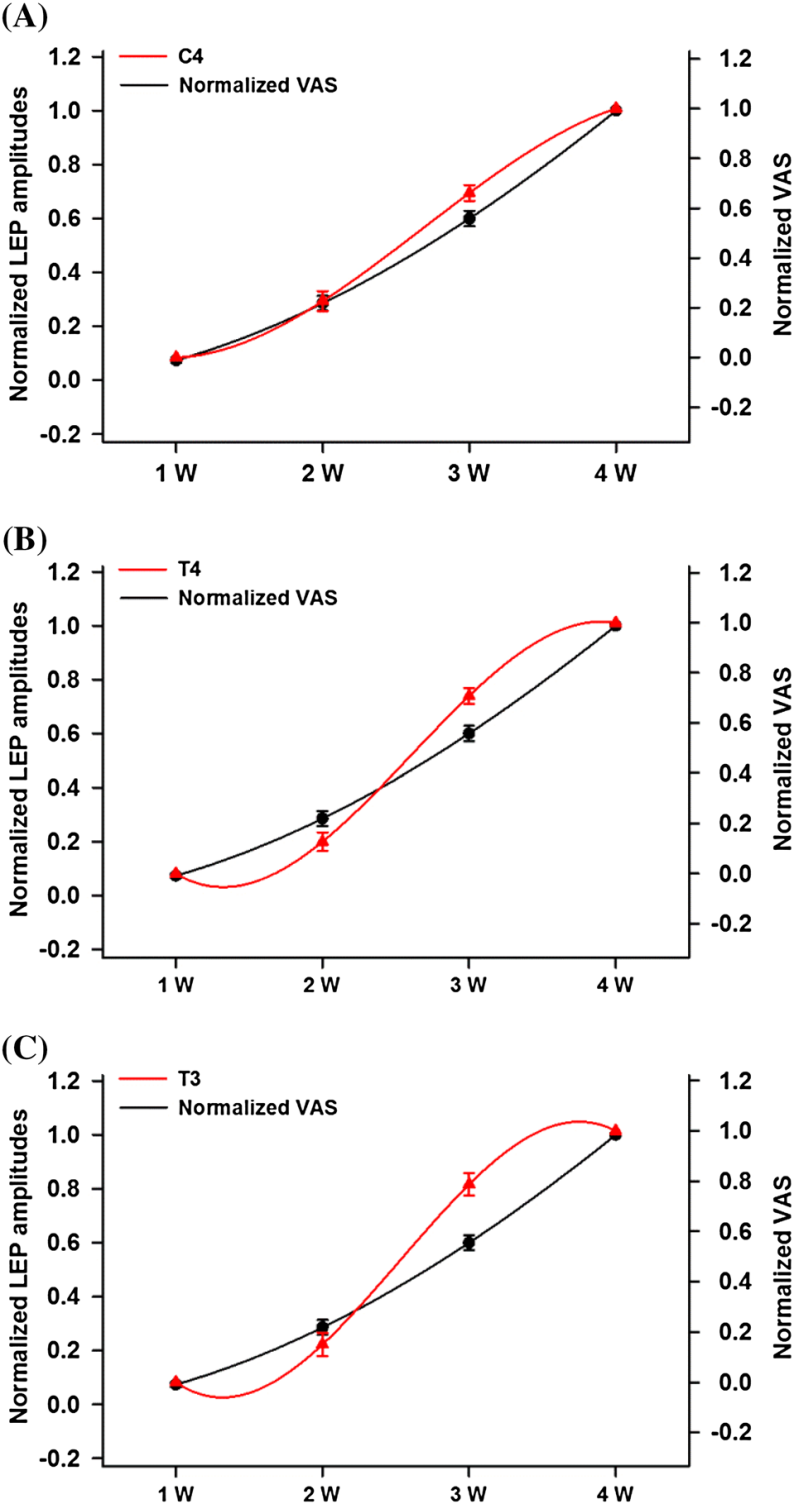
Stimulus–response *curves* and their equation fittings of normalized VAS and normalized LEP amplitude of **(A)** C4, **(B)** T4, and **(C)** T3 leads. Polynomial function fitting is used for changes of normalized LEP amplitudes of C4, T3, and T4 leads. Exponential function fitting is used for VAS changes. Stimulus–response *curve* pattern of C4 LEP is approximate to that of VAS. S-shaped stimulus–response function can be clearly seen in response of T4 and T3 leads

The relation between stimulus intensity and response amplitude revealed fundamental differences in selected channels (Figs. 1, 4). In LEPs of the C4 channel near the cS1, increasing stimulus intensities produced continuously increasing amplitude that closely resembled the exponential pattern (R^2^ = 0.898 ± 0.014). The pain rating with VAS had a great similarity with the exponential pattern (R^2^ = 0.957 ± 0.007). The exponential function fitted significantly better to the individual stimulus–response functions in the C4 lead than to the stimulus–response functions in the T4 (R^2^ = 0.867 ± 0.015, *p* < 0.001) and T3 (R^2^ = 0.824 ± 0.024, *p* < 0.001) leads.

When we further examined the stimulus–response patterns of the T3 and T4 leads, the N2–P2 amplitudes were low at subthreshold and threshold intensities, and there was a sharp increase in N2–P2 amplitude at stimuli well above the pain threshold. The stimulus–response pattern was analogous to an S-shaped curve. A polynomial function can approximate either an S-shaped or exponential curve. Thus, a polynomial function was fitted to the stimulus–response functions to describe the differences in selected leads (Fig. 4). The coefficients of the normalized peak amplitude of C4 (b_2_ = −0.63 ± 0.31, b_3_ = 0.28 ± 0.2) were not significantly different from that of the VAS ratio (b_2_ = −0.0098 ± 0.187, *p* = 0.118, b_3_ = −0.0183 ± 0.115, *p* = 0.108), but significantly different from that of T4 (b_2_ = −1.91 ± 0.38, *p* = 0.013, b_3_ = 1.06 ± 0.24, *p* < 0.001) and T3 (b_2_ = −2.25 ± 0.45, *p* = 0.005, b_3_ = 1.25 ± 0.28, *p* < 0.001, Bonferroni corrected).

**Fig. 5:**
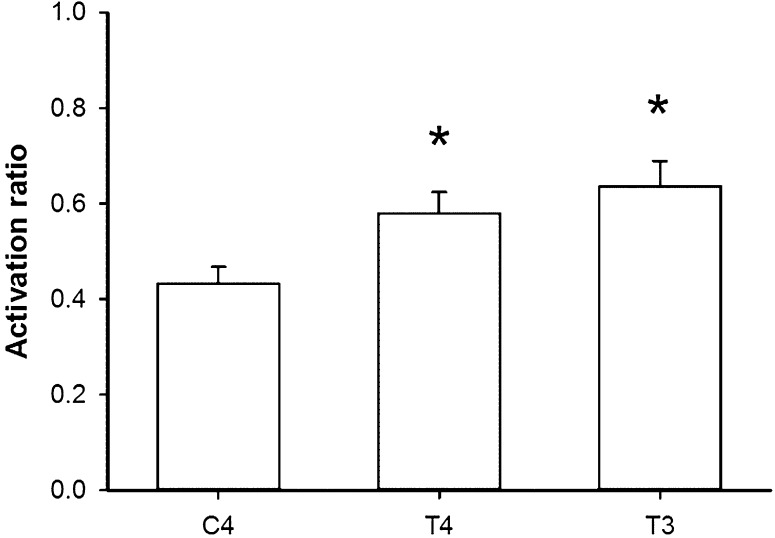
Activation ratio is characterized by change of normalized N2–P2 amplitude between 2- and 3-W stimulation. Activation ratio of C4 lead between 2- and 3-W stimuli is significantly smaller than those of T4 and T3 leads. *p < 0.05 versus C4

Comparison between normalized N2–P2 amplitudes and normalized VAS values in the stimulus–response curve revealed clear differences in the step from 2- and 3-W stimulations. The change of the normalized peak amplitudes is considered as the activation ratio here. The activation ratio of the C4 lead between 2- and 3-W stimuli was significantly smaller than those of the T4 (*p* = 0.003) and T3 (*p* = 0.002) leads (Fig. 5). The results indicate a dramatic increase in the bilateral parasylvian regions in response to laser threshold stimuli. The change in the absolute peak amplitude in T4 (*p* = 0.001) and T3 (*p* = 0.003) leads compared to that of the C4 lead was also significantly different.

## Discussion

In this study, LEPs of C4, T4, and T3 leads, which are located over cS1, cS2, and iS2 areas, respectively, showed significant differences in response to various laser intensities. The LEPs of the C4 and T4 leads started earlier, and either the onset latency or peak latency of the LEPs was almost equivalent in these leads. The LEP amplitudes of these three scalp electrodes increased as stimulation intensity increased. Normalized LEP amplitudes in response to stimulation intensities showed a scalp-lead-dependent pattern. The stimulus–response function of the C4 lead had a high correlation with the pain rating with VAS, with an exponential increasing trend. In contrast, an S-shaped stimulus–response curve was seen in both the T3 and T4 leads. Different stimulus–response patterns for different scalp locations may indicate distinct somatic processes of brain regions. These results suggest that particular scalp channels are able to reflect the functional characteristics of the underlying cortex.

Compared to invasive intraepidermal electrical stimulation [26, 27], noxious laser stimulation is a noncontact stimulus that can be used to elicit nociceptive Aδ or C afferent fibers [16]. Laser stimulation is a non-invasive tool that can be easily applied to a wide body area. Numerous studies have used laser stimulator to investigate the brain processing of pain in healthy subjects using non-invasive EEG or magnetoencephalography (MEG) [18, 25, 28] or in patients using invasive intracranial recordings [29, 30]. Moreover, laser stimulation has been used to evaluate phenomena of hyperalgesia or allodynia in subjects with fibromyalgia [9], migraine [8], stroke [31], or spinal cord injury [32]. Laser stimulation has also been used to evaluate the analgesic effect of drugs [33, 34].

Subjective measures, such as interviews, questionnaires, and quantitative sensory tests, are commonly used in clinics. Objective measures of pain using neuroimaging and EEG/MEG have recently been studied. In general clinical settings, the responses of particular scalp EEG leads, such as Cz or Pz, are commonly used to determine pain-related change [8–10]. The N2–P2 amplitude of LEPs was maximal around the midline region, which is primarily related to the activation of the cingulate cortex. The affective dimension of pain is believed to be related to the cingulate cortex, and discrimination and sensory integration of noxious stimuli are associated with the somatosensory cortex [35]. In the present study, we observed different intensity-related functional characteristics in LEPs of EEG leads with regard to the somatosensory cortex, such as T3/T4 for S2 and C4 for S1. The different response patterns between parietal (C4) and parasylvian (T3/T4) regions are comparable to observations in MEG studies [25, 36] and intracranial recordings of humans [29, 37]. Functional evaluation of pain in the somatosensory cortex based on EEG-lead-related position has also been conducted using transcranial magnetic stimulation (TMS) [38–40]. These results support that EEG leads can reflect the activity of their underlying cortical area and can be used in the clinical assessment of pain attributes.

The C4 lead is over the hand representative area of S1. An earlier contralateral activation in response to laser stimulation was found in the contralateral side (Fig. 2). The LEP of the C4 lead had a trend similar to that of the pain rating with VAS, which behaved as an exponential increasing function in response to stimulus intensity. This linear/exponential increase of the stimulus–response function is consistently seen in S1 either using intracranial electrocorticographic recordings in humans [29, 37, 41] or intracranial single- or multi-unit recordings in animals [42–44]. A lesion in S1 causes problems in sensory discrimination in humans [2, 3] and rats [4]. Moreover, TMS applied over the hand representative area of S1 affects pain perception [38, 40]. A somatotopic map for pain has been delineated in S1 [27], which is believed to process the discrimination of the stimulus location. These data suggest a role of S1 in the discrimination of the intensity and location of a stimulus. The C4 lead of scalp EEG can thus be used to reflect the functional characteristics of S1.

Inconsistent results obtained using various recording techniques have been reported regarding the involvement of S1 in response to nociceptive stimuli in humans. For example, about 50–75 % of neuroimaging studies detected S1 activity in response to nociceptive inputs [5, 6]. In contrast, there are consistent findings regarding S1 involvement in pain processing obtained using electrocorticographic analysis of the subdural grid array [27, 29, 30]. In the equivalent dipole analysis of LEPs, there are inconsistent findings on the parietal dipole [13, 18–21]. The parietal dipole can be contributed by areas 1 or 2 of S1 [13] and even extended to M1 [5, 45]. The present study obtained a widespread distribution of the parietal dipole, with 45 % of subjects showing equivalent current sources in the S1/M1 area. In equivalent dipole analysis, scalp LEPs are influenced by low-pass and spatial filtering at the scalp, skull, and cerebrospinal fluid, by large interelectrode distances, and by possible muscle and blink artefacts [29]. In fact, there is no ideal head model for the dipole source analysis approach. These exogenous sources of interference may affect the dipole modeling results of scalp LEPs. Thus, the present study used scalp EEG leads instead of dipole sources.

The LEPs of T3/T4 leads, near the parasylvian region of S2 and the insula, revealed an S-shaped stimulus–response pattern. The S-shaped stimulus–response pattern is consistent with findings obtained using MEG [25, 36] and intracranial subdural recordings [37]. The S-shaped response cannot fully reflect the pain rating in the present study and previous studies [36, 37]. S2 had a higher threshold than that of S1 and was unable to reflect pain ratings in response to noxious laser stimuli in a single-trial fMRI study [46]. Pain sensitivity is altered by a lesion in the parasylvian cortex [2] and TMS interference over S2 [39]. These data may indicate a discrimination role of S2 in pain processing. In the present study, we found dipole sources in the parasylvian regions of S2 and the insula in 80 % of subjects. The S-shaped stimulus–response curve of S2 remarkably differs from the linear stimulus–response curve of the insula [37, 46]. There are two kinds of neuron, namely nociceptive-specific and wide-dynamic-range neurons, in the S2–insula region of primates [47, 48]. The multi-type neuron population of S2 may indicate a convergent process for different exogenous inputs. These results may also suggest the role of sensory integration for the parasylvian area [35].

The present study observed no significant difference between the dorsal parietal region of the scalp C4 lead and the parasylvian region of the scalp T4 lead in terms of onset latency and peak latency of LEPs (Table 1). Our data may suggest parallel processes in these two regions in response to laser stimulation. There are inconsistent findings for the contralateral cerebral process of pain, with either the serial process of S1 leading [18, 45], the serial process of the parasylvian region leading [19, 49], or parallel processes [13, 28]. These inconsistent findings may arise from different tools used for recording and analysis, different signal-to-noise ratios, or different sample sizes.

The latency difference of parasylvian activities between hemispheres is ~20 ms (Table 1). The interhemispheric delay is comparable with that reported in previous studies [28, 45, 49]. This delay may be due to parallel ascending nociceptive pathways [35] and a possible contribution through interhemispheric conduction of the corpus callosum.

Scalp EEG primarily reflects neural activity from the upper layer of the cortex. Scalp EEG has limitations of low spatial resolution and sensitivity compared to those of MEG or intracranial recordings. The present study selected the C4, T4, and T3 leads, which were close to the right postcentral gyrus and the right and left sylvian fissure, respectively [22], to reflect cortical responses of laser stimuli. The activities of these leads showed a great correlation with pain processes of different cortical regions in terms of the stimulus–response pattern, onset and peak latency, and dipole analysis. Scalp EEG may provide an easy and simple way to measure pain-related function.

Numerous studies have applied lasers to investigate the pain process in either healthy subjects or patients [9, 17, 18]. The present study found that LEPs of scalp EEG reflect distinct brain functions in response to various laser intensities. Therefore, the present study provides additional results for further analysis of big data or meta-analysis, which can determine the discrimination characteristics of the cortex. Moreover, our results can strengthen the application of the recording of scalp EEG leads in other research fields, such as perception of visual or auditory stimulation, cognition of attention, or memory processing.

The scalp EEG leads showed distinct LEPs with regard to various intensities in different scalp locations. A potential limitation in the present study is that the scalp EEG leads have a limited spatial resolution, and EEG has a lower signal-to-noise ratio compared to that of intracranial recording. Intracranial recording is a better way to explore subtle brain changes and to understand different aspects of pain processes. Accordingly, intracranial recording should be used to verify the findings of this study.

## Conclusions

The present results used scalp leads to characterize a somatic process in terms of the latency and amplitude of LEPs and stimulus–response curves. Our data suggest the primary somatosensory cortex-related lead (C4) may reflect pain rating function and parasylvian-related leads (T3/T4) may indicate pain detection/integration function. Accordingly, scalp leads can be used to conveniently measure for the pain process in clinical settings.
